# Supplementary material for: Optimization of anther culture of awnless triticale
Source: PeerJ. 2025 Sep 30;13:e19951. doi: 10.7717/peerj.19951 (PMC12493755; doi:10.7717/peerj.19951)
Supplement: Supplemental Information 2 [file peerj-13-19951-s002.docx]

**CHB medium formulation**

| **Composition** | **Chemical formula** | **Content (mg/L)** |
| --- | --- | --- |
| Calcium chloride dihydrate | CaCl_2_·2H_2_O | 83.0 |
| Cobalt chloride hexahydrate | CoCl_2_·6H_2_O | 0.0125 |
| Copper sulfate pentahydrate | CuSO_4_·5H_2_O | 0.0125 |
| Boric acid | H_3_BO_3_ | 5.0 |
| Potassium phosphate monobasic | KH_2_PO_4_ | 200.0 |
| Potassium iodide | KI | 0.4 |
| Potassium nitrate | KNO_3_ | 1415.0 |
| Magnesium sulfate | MgSO_4_·7H_2_O | 93.0 |
| Manganese sulfate tetrahydrate | MnSO_4_·4H_2_O | 5.0 |
| Ethylenediaminetetraacetic acid monosodium ferric salt | C_10_H_12_FeN_2_NaO_8_ | 32.0 |
| Sodium molybdate dihydrate | Na_2_MoO_4_·2H_2_O | 0.0125 |
| Ammonium sulfate | (NH_4_)_2_SO_4_ | 232.0 |
| Zinc sulfate heptahydrate | ZnSO_4_·7H_2_O | 5.0 |
| Glycine | C_2_H_5_NO_2_ | 1.0 |
| Glutamine | C_5_H_10_N_2_O_3_ | 1000.0 |
| Inositol | C_6_H_12_O_6_ | 300.0 |
| Vitamin C | C_6_H_8_O_6_ | 0.5 |
| Vitamin B1 | C_12_H_17_ClN_4_OS | 2.5 |
| Vitamin B6 | C_8_H_11_NO_3_ | 0.5 |
| Vitamin H | C_10_H_16_N_2_O_3_S | 0.25 |
| Nicotinic acid | C_6_H_5_NO_2_ | 0.5 |

**MS medium formulation**

| **Composition** | **Chemical formula** | **Content (g/L)** |
| --- | --- | --- |
| MS medium | - | 4.74 |
| sorbitol | C_6_H_14_O_6_ | 10.0 |
| sucrose | C_12_H_22_O_11_ | 30.0 |
| agar | (C_12_H_18_O_9_)_n_ | 7.0 |
