# Supplementary material for: Optimization of anther culture of awnless triticale
Source: PeerJ. 2025 Sep 30;13:e19951. doi: 10.7717/peerj.19951 (PMC12493755; doi:10.7717/peerj.19951)
Supplement: Supplemental Information 3 [file peerj-13-19951-s003.docx]

**Highlights:**

1.The best low-temperature pretreatment days and hormone concentration for anther cultures of awnless triticale were determined in this manuscript.

2.The best low-temperature pretreatment days for anther cultures of awnless triticale was 15 d.

3. The best hormone concentration for anther cultures of awnless triticale was 1.5 mg/L 2,4-D + 1.5 mg/L KT.

4. Among the 36 regenerated green plants that were grown in the field, The 9 DH1 plants reached the standard of awnless triticale, which could be used as materials for breeding new awnless triticale varieties.
