# Supplementary material for: Optimization of anther culture of awnless triticale
Source: PeerJ. 2025 Sep 30;13:e19951. doi: 10.7717/peerj.19951 (PMC12493755; doi:10.7717/peerj.19951)
Supplement: Supplemental Information 4 [file peerj-13-19951-s004.doc]

Reviewer

1 please supply a file with the translations for the non-English text (for example: 有效分蘖数 in the "Agronomic traits of 13 DH1" tab) used in: FILE-NAME-HERE. The translations must be in a DOC or TXT file.

Response

Thanks for your suggestion.

（1）株高 was plant height (cm) in the ‘Agronomic traits of 13 DH1’ tab and Rawdata.xlsx. Plant height was that distance from the ground to the top of the spike, but excluding the awn, which was measured using a ruler.

（2）有效分蘖 was effective tiller in the ‘Agronomic traits of 13 DH1’ tab and Rawdata.xlsx. Effective tiller was that the height of triticale branches was higher than 50 cm. 有效分蘖数 was number of effective tiller, which was that the number of triticale branches with a height greater than 50 cm.

（3）顶芒was tip awn (mm). Tip awn length was the length of the longest awn at the top of the spike, which was measured using a vernier caliper.

（4）侧芒was side awn (mm). Side awn length was the length of the longest awn of the middle spikelet on both sides of the spike, which was measured and then the average value was calculated.

（5）穗长was spike length (cm). Spike length was that length from the basal spikelet to the tip of the spikelet, but excluding the awn.

（6）小穗数was number of spikelets, which was number of fertile and sterile spikelets.

（7）穗粒数was number of grains per spike. Number of grains per spike was that number of grains in a triticale spike.

（8）穗粒重was grain weight per spike (g). Grain weight per spike was the weight of grain in a triticale spike, which determined using an electronic balance.

（9）绿苗分化率was green plantlet differentiation frequency (DFG). DFG (%) = Number of green plantlets/Number of calli × 100.

（10）白苗分化率was albino plantlet differentiation frequency (DFA). DFA (%) = Number of albino plantlets/Number of calli × 100.

（11）绿苗产率was green plantlet production (PRG). PRG (%) = Number of green plantlets/Number of anthers used for the inoculation × 100.

（12）白苗产率was albino plantlet production (PRA). PRA (%) = Number of albino plantlets/Number of anthers used for the inoculation × 100.

（13）植株再生率was plant regeneration rate (PRR). PRR (%) = Total number of plantlets/Number of anthers used for the inoculation × 100.

（14）描述: it means description.

（15）均值的 95% 置信区间: it means 95% confidence intercal for mean.

（16）均值 was average value.

（17）标准差was standard deviation(S.D.).

（18）标准误was standard error(S.E.).

（19）上限was upper limit.

（20）下限was lower limit.

（21）极大值was maximum value.

（22）极小值was minimum value.

（23）组内was within-group.

（24）组间was between-group.

（25）总数was total.

（26）单因素方差分析was One-way ANOVA.

（27）平方和was sum of square.

（28）均方was mean square.

（29）显著性was significance.

（30）序号 was serial number.

（31）alpha = 0.05 的子集: it means subset of alpha = 0.05.

（32）将显示同类子集中的组均值: it means that displaying group means within homogeneous subsets.

（33）a. 将使用调和均值样本大小 = 4.000: it means that the harmonic mean sample size will be used = 4.000.
